# Robotic-Assisted Versus Conventional Total Knee Arthroplasty: A Systematic Review and Meta-Analysis of Randomized and Quasi-randomized Controlled Trials Evaluating Perioperative and Radiographic Outcomes

**DOI:** 10.7759/cureus.106394

**Published:** 2026-04-03

**Authors:** Mohammad Ahmed Idrees, Shamaila Ibrahim, Muhammad Ibrahim

**Affiliations:** 1 Neurosurgery, Royal Sussex County Hospital, Brighton, GBR; 2 Obstetrics and Gynecology, King's College Hospital, London, GBR; 3 General Surgery, Barts Cancer Institute, Queen Mary University of London, London, GBR; 4 General Surgery, Ahmadiyya Muslim Hospital, Daboase, GHA

**Keywords:** conventional total knee arthroplasty, manual total knee arthroplasty, perioperative outcome, radiographic outcomes, robotic assisted total knee arthroplasty, robotic knee surgery, systematic review and meta-analysis, total knee arthroplasty

## Abstract

Robotic-assisted total knee arthroplasty (RATKA) has garnered attention in the field of orthopedic surgery. It has been developed to improve surgical precision and prosthesis alignment in comparison to conventional total knee arthroplasty (CTKA). It utilizes advanced robotic workflow systems as opposed to manual jig-based techniques. This review evaluated perioperative and radiographic outcomes to assess the overall safety and effectiveness of RATKA with CTKA.

A comprehensive search for randomized and quasi-randomized control trials was conducted across 3 databases, PubMed/MEDLINE, Cochrane Library and Embase, from 1st January 2021 to 1st January 2026. Studies were chosen that compared RATKA to CTKA, where the primary indication was knee osteoarthritis. The primary objectives were operative time, length of stay, adverse events and blood loss. The secondary objectives were hip-knee-ankle (HKA) angle and absolute deviation of HKA angle from 180° (ΔHKA). The review was conducted in accordance with the Preferred Reporting Items for Systematic Reviews and Meta-Analyses guidelines (PRISMA). Risk of bias was assessed using the Cochrane risk of bias tool (RoB 2). Quantitative analysis performed using the RevMan 5.4 software package. Results were presented using mean difference (MD), 95% confidence interval (CI), and risk ratio (RR).

A total of 12 controlled trials were identified that met the inclusion criteria, with a total of 2269 participants. Meta-analysis revealed a statistically significant difference in operative duration, with RATKA taking longer than CTKA (MD = 23.81, 95% CI = 13.39 to 34.24, P <0.00001). There were no statistically significant differences in hospital stay (MD = 0.11, 95% CI = -0.19 to 0.42, P = 0.47), intraoperative blood loss (MD = 9.00, 95% CI = -9.46 to 27.46, P = 0.34) and adverse events (RR = 0.80, 95% CI = 0.54 to 1.18, P = 0.28). However, a statistically significant difference was identified in postoperative mechanical alignment, favoring RATKA. Postoperative HKA angle (MD: 0.71°, 95% CI: 0.43 to 1.00, P <0.00001) and absolute deviation from 180° ΔHKA (MD = -1.33, 95% CI -2.12 to -0.55, P = 0.009).

RATKA is associated with longer operating times but is associated with improved mechanical alignment. The intraoperative blood loss, length of hospitalization and complications were comparable to CTKA. Considering these findings, further studies are required to assess the long-term implications and clinical benefits of RATKA.

## Introduction and background

Over the past decade, the global burden of knee osteoarthritis has risen significantly. In 2021, the number of cases of knee osteoarthritis was estimated to be approximately 374 million, an increase of 234% since 1990. This has been in part due to rising population numbers, increased age and obesity rates [1]. As such, it presents a burden upon the healthcare system with high associated costs and resource utilization.

Total knee arthroplasty (TKA), a surgical procedure in which the knee joint is replaced with a prosthetic implant, has become one of the most effective and safe surgical procedures for the treatment of moderate to severe knee osteoarthritis [2]. Considering the rising disease burden, it is imperative that great emphasis be placed on optimization of surgical techniques that improve precision and workflow efficiency.

Conventional total knee arthroplasty (CTKA) utilizes jig-based instruments to guide bone resections and shape the knee joint for implanting the prosthesis. The jigs used are standardized with respect to the prosthesis utilized for implant. As such, bone resections can be imprecise and less accurate, owing to variation in knee anatomy and the overall accuracy that can be achieved by hand [2,3]. This variability may affect the position of the implant, which may have an impact on prosthesis longevity and possibly functional outcomes [2].

Robotic-assisted total knee arthroplasty (RATKA) is a relatively new technology that uses computer-assisted planning and robotic guidance to improve the accuracy of bone resections and implant position. This was developed to address some of the issues present with contemporary jig-based methods. As a result, several robotic platforms have emerged, manufactured by various providers. Examples of such robotic platforms are Mako by Stryker or NAVIO by Smith & Nephew [3].

Whilst numerous systematic reviews have compared the two techniques, they often include non-randomized studies with less analytical rigor and older robotic platforms [4]. The primary focus of previous reviews has been directed towards patient-reported outcomes and postoperative radiographic alignment. In comparison, significantly less analysis has been carried out for key perioperative outcomes. Perioperative metrics influence resource utilization, surgical efficiency and often institutional decision making. They may also serve as indicators of patient safety and recovery [5]. Furthermore, given the rapid evolution of robotic platforms and technological advancements, prior work may not accurately reflect current practice [6].

The primary aim of this systematic review and meta-analysis is to provide an up-to-date synthesis of key perioperative outcomes and postoperative radiographic outcomes. This is achieved by analyzing data available from randomized and quasi-randomized controlled trials published within the last five years (1st January 2021- 1st January 2026), comparing RATKA to CTKA.

The primary outcomes assessed include: operative time, blood loss, hospital stay and adverse events, between the two TKA methods. The secondary outcomes assessed are the postoperative radiographic outcomes. These are the postoperative Hip-Knee-Ankle (HKA) angle, a radiographic measure of lower limb mechanical alignment, and absolute deviation of the postoperative HKA angle from 180° (ΔHKA).

## Review

Methods

This systematic review and meta-analysis were conducted in accordance with the Preferred Reporting Items for Systematic Reviews and Meta-Analyses (PRISMA) guidelines [7] and the Cochrane Handbook for systematic reviews and meta-analysis [8].

Eligibility Criteria

A structured framework was established to define the inclusion and exclusion criteria. Randomized and quasi-randomized trials were included if they met all other predefined eligibility criteria for inclusion and their methodological limitations were accounted for during the risk-of-bias assessment. Table 1 summarises the inclusion and exclusion criteria in a PICOS format.

**Table 1:** PICOS framework for inclusion and exclusion criteria. TKA: Total Knee Arthroplasty; HKA: Hip-Knee-Ankle angle; ΔHKA: Absolute deviation from 180°; CT: Computed tomography

PICOS	Inclusion criteria	Exclusion criteria
Population	Adult patients aged above 18 undergoing primary TKA for knee osteoarthritis	Patients undergoing TKA where the primary indication is not knee osteoarthritis
Intervention	Robot-assisted TKA using any platform and workflow	Unicompartmental, bicompartmental or revision TKA
Control	Conventional or manual jig-based TKA using any mechanical instrumentation	Computer navigated TKA.
Outcomes	Studies reporting any one of the outcomes in a reportable format. Perioperative: blood loss, operating time, adverse events, hospital stay. Radiographic outcomes: HKA angle, ΔHKA.	Studies not comparing RATKA to CTKA or utilizing CT imaging for HKA angle acquisition.
			Study	Randomized and quasi-randomized controlled trials published in English between 1st January 2021 to 1st January 2026.	Non-randomized studies, conference abstracts, unpublished data or studies not available in English.

Search Strategy

A systematic literature search was performed across the online databases: PubMed/MEDLINE, Embase and Cochrane Library. The search covered studies from 1st January 2021 to 1st January 2026. The final search was conducted on 10th February 2026. Search strategies were developed using a combination of medical subject headings (MeSH) terms and free text related to robotic total knee arthroplasty and conventional total knee arthroplasty. Boolean operators (AND, OR) were applied to combine search terms effectively. Different search combinations were used to maximize sensitivity and identification of relevant studies.

A representative search strategy for PubMed/MEDLINE is as follows: (“knee arthroplasty” OR “total knee replacement”) AND (“robotic assisted” OR “robotic”) AND (“manual” OR “conventional”). The search strategy was adapted appropriately for Embase and the Cochrane Library based on database-specific indexing. In addition, Clinical trial registries and a manual screen of the reference list of eligible studies were also performed.

The full database-specific search strategies can be made available from the corresponding author upon reasonable request. 

Study Selection and Data Collection

Two reviewers manually screened titles and abstracts of retrieved results to assess eligibility based on the inclusion and exclusion criteria. Full text article were then reviewed for eligibility. Any disagreements were resolved through discussion, and when consensus was not reached, a third reviewer was consulted.

Data extraction was performed manually and placed into an Excel sheet and a Word document table. The extracted data included the first author's surname, year of publication, sample sizes of respective cohorts and outcome data. This included the mean and standard deviation for continuous variables or the number of events for categorical data, where applicable.

Outcomes

Data were extracted from each selected study for the primary and secondary outcomes. Primary outcomes included operative time (minutes), blood loss (milliliters), hospital stay (days) and adverse events (number of events). Secondary outcomes included postoperative HKA angle and absolute deviation of HKA angle from 180° (ΔHKA).

Operative time was defined as either skin incision to skin closure or total theatre time, depending on study reporting. Blood loss was measured directly or estimated using standardized calculations [9]. Adverse events were counted as the number of events that were assessed across the duration of the specified follow-up period of the study. Given the directional complexity of interpreting raw HKA angle values, absolute deviation from 180° (ΔHKA) was also analyzed as a direction-independent measure of alignment accuracy. 

Whilst outcome definitions varied across studies, data were extracted consistently and pooled using standardized statistical approaches. This variability may contribute to heterogeneity and, therefore, the results were interpreted with caution. Table 2 summarises the primary and secondary outcomes.

**Table 2:** Primary and secondary outcomes assessed in the review. Gross's formula is a common method used for estimating blood loss [9]. HKA: Hip-Knee-Ankle angle.

Primary outcomes	Secondary outcomes
Operative time, measured in minutes (min) and assessed as a continuous variable. Can be defined as skin incision to skin closure or total theatre time.	HKA angle measured in degrees (°) and assessed as a continuous variable. The HKA angle represents the mechanical axis of the lower limb. Normal HKA angle is defined as 180° ± 3, with ideal alignment defined as 180°. Measured using plain radiographs postoperatively. HKA angle is a direction-dependent metric.
		Estimated Blood loss, measured in milliliters (ml) and assessed as a continuous variable. Can be estimated using suction devices or theoretically calculated from standardized formulas
Hospital stays are measured in days and assessed as a continuous variable		Absolute deviation of the postoperative HKA angle from the ideal alignment 180° (ΔHKA). Calculated from measurements obtained from plain radiographs. This metric provides a direction independent measure of alignment accuracy.
Adverse events or complications counted as the number of events and assessed as a dichotomous variable. Includes, medical and surgical complications extracted across the full reported follow up period of each study.		

Risk of Bias Assessment

Risk of bias assessment was done using Cochrane Risk of Bias 2 (ROB 2) Excel tool (version 9, 2019). Assessment was carried out at an outcome level across all 5 domains. Two reviewers evaluated each domain, and the results were discussed to reach consensus. Disagreements were resolved by consulting a third reviewer. The signaling questions within the ROB 2 tool were used to guide judgments, and the algorithm provided an overall risk of bias for each outcome [10]. Traffic-light plots were generated using ROBVIS for visual representation [11].

Data Synthesis

Statistical analysis was performed using the Review Manager software (REVMAN 5.4) [12]. Effect measures for continuous variables were computed using mean differences (MD) and 95% confidence intervals (CI). For dichotomous outcomes, risk ratios (RR) with 95% CI were calculated.

The evaluated outcomes were pooled using the random-effects model to account for anticipated variation in clinical study design and methodology. The method used for continuous variables was Inverse variance, and for dichotomous variables was Mantel-Haenszel. Heterogeneity was measured using the Chi-squared (χ²) test, I-squared (I2) and Tau-squared (τ2). I2 values were interpreted by following the guidance in the Cochrane handbook. 0% to 40% low heterogeneity; 30% to 60% moderate heterogeneity; 50% to 90%: Substantial heterogeneity; 75% to 100% considerable heterogeneity [8]. The overall effect of the measure for each outcome was evaluated with a Z-test. To visually represent the data, forest plots were generated for all pooled outcomes.

In studies where multiple robotic intervention arms were compared to a single manual control group, the robotic arms were combined into a single intervention group to avoid double-counting. This was done by calculating pooled means and standard deviations using weighted methods recommended by the Cochrane guidance. In studies with mutually exclusive subgroups comparing RATKA to CTKA, each subgroup was treated as a separate dataset and labeled accordingly to avoid data exclusion [8].

Where feasible, sensitivity analysis was carried out to explore potential sources of heterogeneity. This was done by restricting pooled analysis to studies with comparable study protocols, image acquisition techniques, follow-up timepoints or robotic platforms. Assessment of publication bias was planned using funnel plots, but this was limited by the number of included studies and considerable heterogeneity of observed outcomes, reducing the reliability of such analysis.

Results

Study Selection

**Figure 1:**
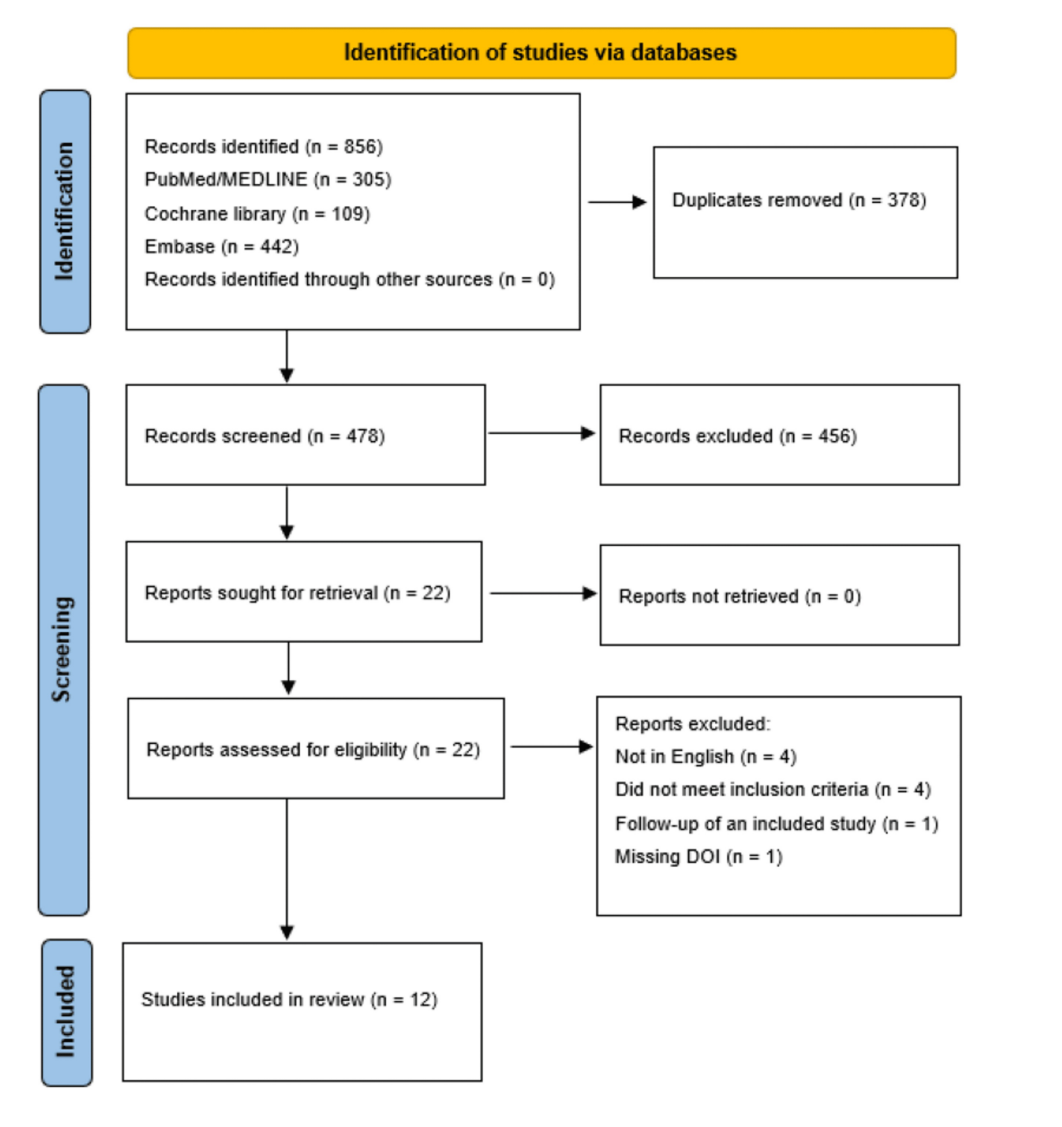
PRISMA flow diagram of the study selection process and how many studies were included in the review. Filled in from guidelines detailed in Page et al. [7].

Across three databases, a total of 856 results were identified: PubMed/MEDLINE (n = 305), Cochrane Library (n = 109) and Embase (n = 442). No additional eligible records were identified through other sources, including trial registry searches and manual reference screening (n = 0). After the removal of duplicates, the remaining 478 results were screened based on titles and abstracts. 22 studies remained for full-text review and were assessed for eligibility based on predefined criteria. Of the 22 studies, 10 were excluded for various reasons. Four studies were not available in English. One study was a longer-term follow-up performed by the same authors already included in the review. Four studies did not meet the inclusion criteria regarding imaging protocols and outcomes of interest. One study had an incorrect digital object identifier. Finally, 12 studies were deemed to meet the inclusion criteria for population, intervention and outcomes of interest. The PRISMA flow diagram is detailed below in Figure 1.

Study Characteristics

Across the 12 included studies, there were a total of 1178 patients in the RATKA group and 1091 patients in the CTKA group [13-24]. The average age of patients across the 12 studies in both groups was comparable, and there was a similar surgical technique being utilized for manual TKA. The primary indication for TKA across all the trials was knee osteoarthritis. There were a variety of robotic platforms being used across the studies, which included NAVIO (4), CORI (2), EPMEDBOT (1), MAKO (1) and YUANHUA‐TKA robot (2). Two papers stated that the company manufactures the robot platform, and one study did not specify the robotic platform used. The baseline characteristics are summarized in Table 3.

**Table 3:** Baseline characteristics of each included study. Representing the number of patients in each group, the average age of the patients, the robotic platform utilized and the country where the study was performed. Age is presented as the mean age and standard deviation. RATKA: Robotic-assisted total knee replacement; CTKA: Conventional total knee replacement. MAKO, NAVIO, CORI, EPMEDBOT and YUANHUA-TKA represent names of the robotic platform used. Beijing AKEC Medical and Hangzhou Jianjia are the names of the companies manufacturing the robot system.

Baseline characteristics of analysed studies						
Author	Age RATKA	Age CTKA	Robotic platform utilized	RATKA (n)	CTKA (n)	Country study took place
Geng et al. 2024 [13].	68.10 ± 5.00	67.40 ± 5.50	Beijing AKEC Medical- OP‐RKL22	63	62	China
Ren et al. 2025 [14].	66.80 ± 5.80	66.80 ± 5.1	EPMEDBOT	32	34	China
Migliorini et al. 2025 [15].	66.70 ± 8.40	67.00 ± 8.10	CORI	500	500	Germany
Skaden et al. 2025 [16].	65.70 ± 3.70	67.70 ± 3.90	NAVIO	26	25	Norway
Bollars et al. 2025 [17].	64.50 ± 8.90	65.80 ± 8.60	NAVIO	89	90	Belgium
Tian et al. 2023 [18].	68.17 ± 7.59	68.84 ± 7.12	Hangzhou Jianjia Robot system	62	61	China
Xu et al. 2022 [19].	64.50 ± 5.30	63.40 ± 7.20	YUANHUA‐TKA	37	35	China
Geng et al. 2025 [20].	67.37 ± 5.15	68.33 ± 5.80	Not specified	73	66	China
Clement et al. 2023 [21].	66.80 ± 8.70	66.70 ± 9.60	MAKO	46	41	United Kingdom
Thiengwittayaporn et al. 2021 [22].	69.00 ± 8.30	69.10 ± 7.30	NAVIO	75	77	Thailand
Adamska et al. 2023 [23].	67.44 ± 7.30	65.00 ± 8.20	NAVIO, CORI	147	68	Poland
Yuan et al. 2024 [24].	65.20 ± 6.40	65.40 ± 8.00	YUANHUA‐TKA	28	32	China

Risk of Bias in Studies

**Figure 2:**
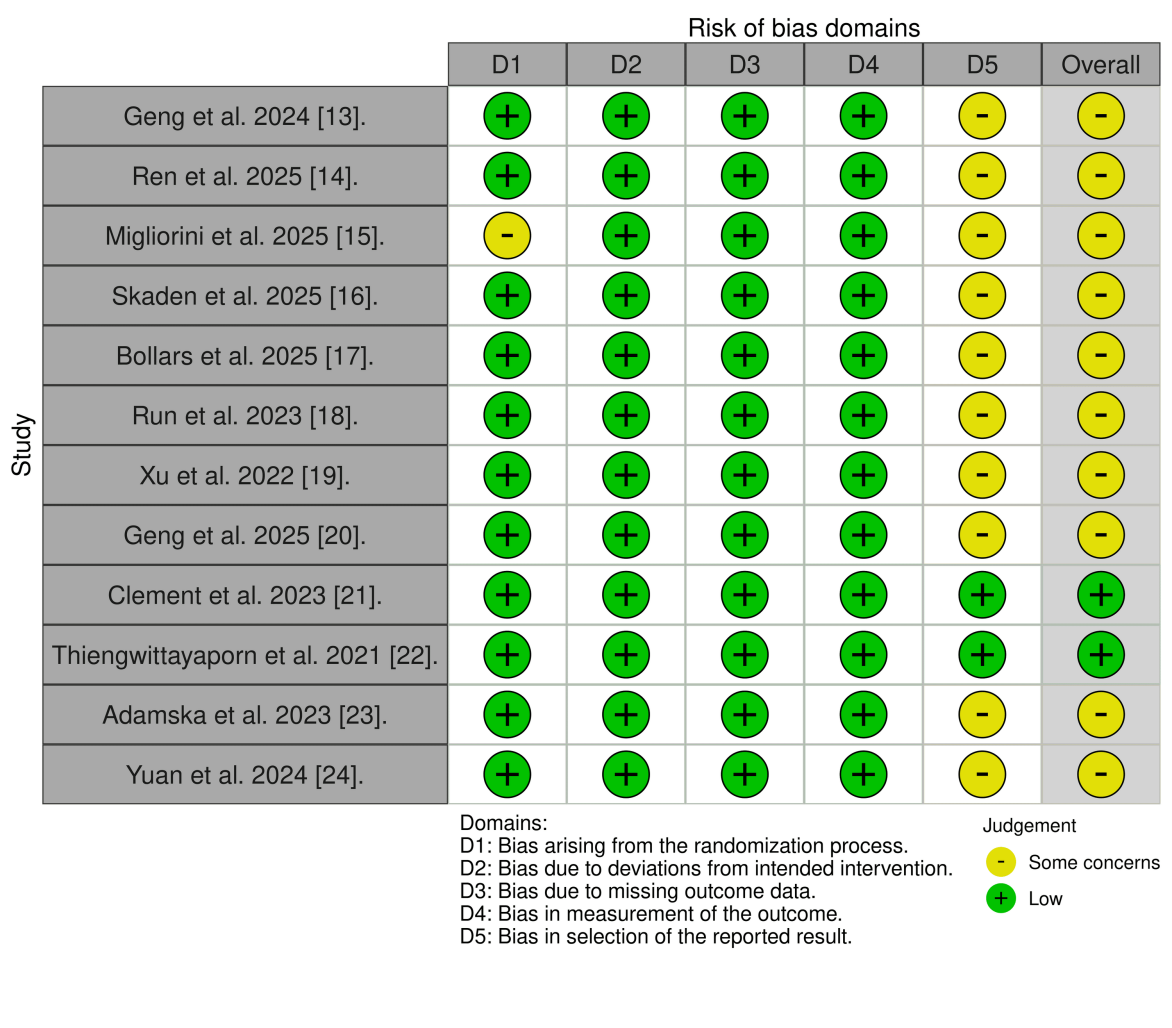
A traffic light plot for the overall risk of bias created for each study based on the Outcome level risk of bias The traffic light plot was created using the robvis tool [11]

The risk of bias was assessed at the outcome level using the ROB 2 Excel tool algorithm and independent reviewer assessment in accordance with Cochrane guidance [7]. Overall study risk of bias was derived from the outcome with the highest risk assessment within each study. An overall risk of bias table was constructed for visual representation. Two studies were judged to be low risk of bias, while the 10 remaining studies were judged to have some concerns. Moreover, none of the included studies were judged to be high risk. All the studies were judged as low risk in Domains 1 to 4. 10 studies demonstrated some concerns in Domain 5, primarily due to a lack of a prespecified study protocol detailing the outcomes being assessed. A traffic light plot of the overall risk of bias is shown in Figure 2.

Operative Time

**Figure 3:**
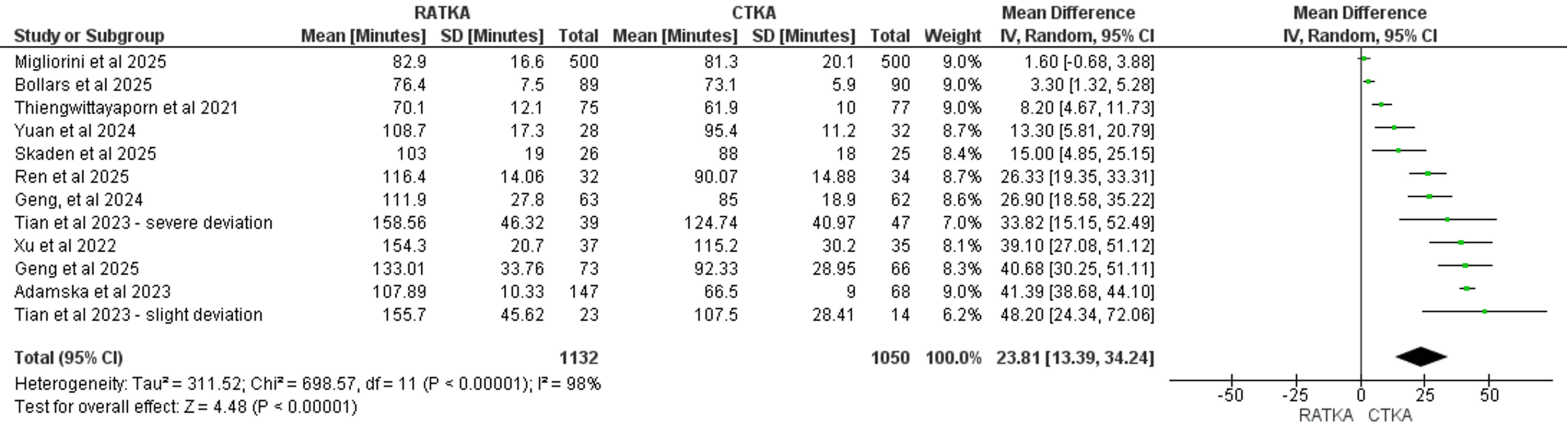
Forest plot representation of Operative time measured in minutes. Studies that reported operative time: Geng et al. 2024 [13]; Ren et al. 2025 [14]; Migliorini et al. 2025 [15]; Skaden et al. 2025 [16]; Bollars et al. 2025 [17]; Tian et al. 2023 [18]; Xu et al. 2022 [19]; Geng et al. 2025 [20]; Thiengwittayaporn et al 2021 [22]; Adamska et al. 2023 [23]; Yuan et al. 2024 [24]. Tian et al. split their data set, and as such was labeled as slight deviation and severe deviation.

Eleven studies reported operative time with a total of 1132 patients in the RATKA group and 1050 patients in the CTKA group. Pooled analysis demonstrated that RATKA was 23.81 minutes longer than CTKA (MD = 23.81, 95% CI = 13.39 to 34.24, P <0.00001). The results were statistically significant. There was considerable statistical heterogeneity across the studies (χ² = 698.57, I2 = 98%, P <0.00001). The forest plot representation of the pooled analysis is displayed in Figure 3.

A sensitivity analysis was performed by including studies that only utilized the NAVIO robotic system. Studies that performed multi-arm analysis between RATKA and CTKA utilizing the NAVIO system were excluded from the sensitivity analysis. The results were statistically significant and consistent with the pooled meta-analysis, with RATKA taking longer than CTKA by 7.16 minutes (MD 7.16, 95% CI 2.1 to 12.30, P = 0.006). Statistical heterogeneity was still considerable, although less than in the pooled analysis. (χ² = 9.64, I2 =79%, P = 0.008).

Blood Loss

**Figure 4:**
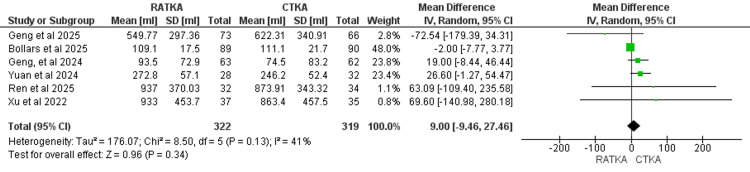
Forest plot representation of data reported for blood loss across included studies measured in millilitres. Studies reporting blood loss: Geng et al. 2024 [13]; Ren et al. 2025 [14]; Bollars et al. 2025 [17]; Xu et al. 2022 [19]; Geng et al. 2025 [20]; Yuan et al. 2024 [24].

Blood loss was reported across six studies comprising 322 patients in the RATKA group and 319 in the CTKA group. The pooled analysis demonstrated a mean difference of 9 ml between RATKA and CTKA, which was not statistically significant (MD = 9.00, 95% CI = -9.46 to 27.46, P = 0.34). Statistical heterogeneity was moderate across the studies (χ² = 8.50, I2 =41%, P = 0.13). The forest plot of the pooled analysis is shown in Figure 4.

Complications or Adverse Events

**Figure 5:**
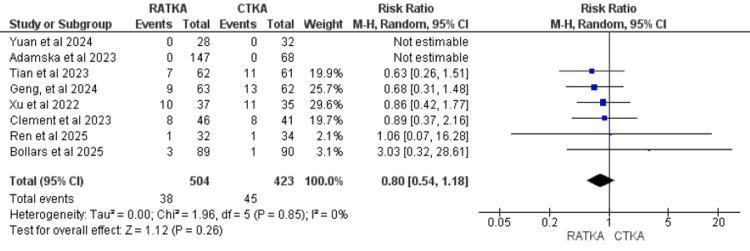
Forest plot representation of Adverse events assessed using risk ratios. Studies that reported adverse events: Geng et al. 2024 [13]; Ren et al. 2025 [14]; Bollars et al. 2025 [17]; Tian et al. 2023 [18]; Xu et al. 2022 [19]; Clement et al. 2023 [21]; Adamska et al. 2023 [23]; Yuan et al. 2024 [24].

Eight studies reported postoperative adverse events, comprising 504 patients in the RATKA group and 423 in the CTKA group. A sum of 38 adverse events occurred in the RATKA group and 45 events in the CTKA group. Pooled analysis demonstrated a RR of 0.80 (RR = 0.80, 95% CI = 0.54 to 1.18, P = 0.28), indicating no statistically significant differences in rates of adverse events between RATKA and CTKA. Statistical heterogeneity was low across the studies (χ² = 1.96, I2 =0%, P = 0.85). The forest plot representation of the analysis is shown in Figure 5.

Hospital Stay

**Figure 6:**
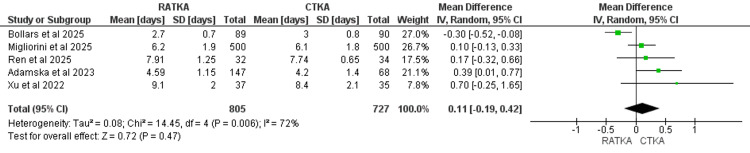
Forest plot representation of hospital stay measured in days. Assessed using the mean difference. Studies that reported hospital stay: Ren et al. 2025 [14]; Migliorini et al. 2025 [15]; Bollars et al. 2025 [17]; Xu et al. 2022 [19]; Adamska et al. 2023 [23].

The duration of hospital stay was reported across five studies with 805 patients in the RATKA group and 727 patients in the CTKA group. Pooled analysis demonstrated a difference of 0.11 days (MD = 0.11, 95% CI = -0.19 to 0.42, P = 0.47). The results indicated there is no statistically significant difference in Hospital stay between the two groups. There was, however, considerable statistical heterogeneity (χ² = 14.45, I2 =72%, P = 0.006). The forest plot of the pooled analysis is shown in Figure 6.

Postoperative HKA Angle

Four studies reported postoperative HKA angle. The total sample size was 215 patients in the RATKA group and 211 patients in the CTKA group. Three studies measured alignment postoperatively at roughly 12 weeks postoperatively using weight-bearing radiographs. One study reported HKA angle 6 weeks postoperatively using a Picture Archiving and Communication System (PACS).

**Figure 7:**
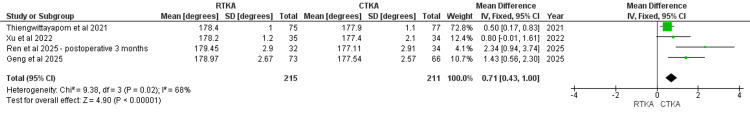
Forest plot representation of postoperative HKA angle. Measured in degrees and assessed using mean difference Studies reporting HKA angle: Ren et al. 2025 [14]; Xu et al. 2022 [19]; Geng et al. 2025 [20]; Thiengwittayaporn et al. 2021 [22]. It is important to note that the mean difference is calculated as the mean values of CTKA subtracted from RATKA. The mean difference in this case is positive, resulting in the forest plot being directed to the right. Better mechanical alignment is assessed with reference to the ideal angle of 180°. The mean HKA values of the RATKA group are closer to 180° as opposed to the CTKA group. As such, the positive mean difference is in favor of the RATKA group.

Pooled analysis demonstrated a mean difference of 0.71°, favoring RATKA (MD = 0.71, 95% CI = 0.43 to 1.00, P <0.00001). Statistical heterogeneity was considerable (χ² = 9.38, I2 = 68%, P = 0.02). The forest plot of the pooled analysis is shown in Figure 7.

Sensitivity analysis was performed by excluding studies that did not define weight bearing status of radiographs or were taken at a different follow-up period. Similar results were obtained with a pooled mean difference of 1.28 degrees favoring RATKA (MD = 1.28, 95% CI = 0.74 to 1.83, p < 0.00001) and a reduced statistical heterogeneity at (χ² = 3.66, I2 =45%, P = 0.16).

Absolute Deviation of HKA Angle (ΔHKA)

**Figure 8:**
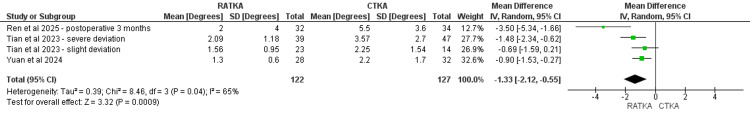
Forest plot of the absolute deviation of the postoperative HKA angle. Studies reporting absolute deviation: Ren et al. 2025 [14]; Tian et al. 2023 [18]; Yuan et al. 2024 [24]. Tian et al. is split into slight and severe deviation groups as was done in the original study. The absolute deviation is calculated by subtracting 180° from the postoperative HKA angle, and then taking the absolute value of that difference. This corrects for directionality observed in assessing postoperative HKA angles on their own, allowing easier interpretation.

Three studies reported absolute deviation of the postoperative HKA angle from 180°. There were 122 patients in the RATKA group and 127 in the CTKA group. Pooled analysis demonstrates a statistically significantly smaller deviation from 180° in the RATKA group compared to the CTKA group (MD = -1.33, 95% CI -2.12 to -0.55, P = 0.009). There was considerable statistical heterogeneity (χ² = 8.46, I2 = 65%, P = 0.04). The forest plot of the pooled analysis is shown in Figure 8.

Discussion

This systematic review synthesized quantitative data from 11 randomized controlled trials and one quasi-randomized controlled trial, focusing on perioperative and radiographic outcomes. We found that, overall, RATKA was associated with significantly longer operative time as compared to CTKA. However, RATKA demonstrated comparable outcomes to CTKA in terms of intraoperative blood loss, length of hospital stay and postoperative complications. In addition, limited analysis suggested that RATKA achieved improved mechanical alignment of the prosthesis as opposed to CTKA.

Robotic platforms have been shown to significantly increase the operating time. The average increase in time was 23.81 minutes as compared to manual techniques. This finding is consistent with previous systematic reviews [25]. An increase in operating time may have the potential to predispose patients to an increased risk of intraoperative complications, although this was not directly assessed in the included studies. In addition, this may imply that the number of procedures performed will be limited, further incurring operational costs [26].

There was significant heterogeneity across studies, which may partly be explained by surgeon experience, variation in robotic platform, learning curves and Setup times. A sensitivity analysis restricted to single-arm studies utilizing the NAVIO robotic system reduced the mean difference to 7.16 minutes, with a corresponding reduction in heterogeneity from 98% to 79%. But heterogeneity remains substantial, and interpretation of the overall effect is still limited by the number of studies present.

No significant difference was observed between RATKA and CTKA in terms of Intraoperative blood loss and hospital stay. These findings have been consistent in previous systematic reviews [26, 27]. Blood loss is an important perioperative parameter that may influence patient recovery and the need for further interventions such as blood transfusions. Similarly, the length of hospital stay may reflect postoperative recovery and may have implications on costs and a significant impact on hospital bed pressures. However, there was significant variation between the studies. This can be attributed to different discharge pathways, postoperative care and surgeon preferences.

The safety profile of RATKA was comparable to the manual techniques in the pooled analysis. There were no statistically significant findings of increased adverse events between the two techniques within the follow-up periods reported in the included studies. This finding was consistent with prior systematic reviews [27]. Safety is one of the metrics that is associated with innovation and prevents exposing patients to harm.

However, there was variation among studies with regards to postoperative follow-up time periods. These variations affected reporting as the complication rates were extracted across the full follow-up period of the included study. As such, longer follow-up periods may capture a greater number of events independent of the intervention, therefore limiting direct comparability between studies.

RATKA was associated with improved mechanical alignment compared to manual jig-based techniques. This was evidenced across the studies reporting postoperative HKA angle and absolute deviation of HKA angle, demonstrating RATKA achieved closer targets to 180° than jig-based techniques. These results have also been found in previous systematic reviews [25,26]. This finding may support the theoretical advantages of robotic systems, yielding improved accuracy and precision of resections as opposed to jig-based platforms.

However, interpretation of raw HKA angles is complex due to directional dependence. The comparison is made with respect to 180°. Values closer to 180° are considered ideal, and the normal range is ± 3° of 180°. As such, it requires careful interpretation. In the analysis, the postoperative HKA angles in the RATKA group were closer to 180° compared to the CTKA group. As a result, the pooled mean difference will be positive, and the forest plot will be to the right side. Therefore, directional improvement should be assessed with respect to 180°. This problem is avoided by using the absolute value of the HKA deviation from 180° instead, which is independent of directionality. However, this metric is less consistently reported and rigorously defined.

In addition, substantial heterogeneity across studies was observed. Heterogeneity arises from differing imaging protocols, radiographic acquisition, imaging modality and the timepoints at which the measurements were acquired. To explore one of the possible sources of heterogeneity, studies that did not acquire images at 12 weeks were excluded. This yielded a reduction in statistical heterogeneity(I2) of 68% to 45%.

The use of randomized and quasi-randomized studies for quantitative analysis, allowing for a higher level of evidence synthesis as compared to observational studies, is one of the strengths of this review. Paired with a comprehensive search strategy, well-defined inclusion and exclusion criteria, alongside careful quantitative analysis, contribute to the robustness of the findings. In addition, utilizing current evidences the relevance of the results to current clinical practice. 

However, several limitations should be acknowledged. Substantial heterogeneity across studies limit precision of pooled estimates and makes it difficult to confidently ascertain accurate comparisons between the two techniques. These differences underpin variations in study design, protocol definitions and different hospital workflow systems. The inclusion of a quasi-randomized study may introduce additional risk of bias, particularly related to the allocation methods. Although this was accounted for in the risk of bias assessment. Considering how fast technological advances take place, it makes it difficult to adequately assess current robotic performance. Moreover, for ΔHKA, very limited datasets were present, resulting in limited data analysis. Finally, there was limited funnel plot analysis, owing to the large heterogeneity and small number of studies analysed reducing the reliability of such analysis.

Despite these limitations, the review highlights key areas for future research. Standardization of outcome definitions, imaging protocols, and follow-up time periods is essential to allow comparability between studies. It also means that key areas of improvement can be tackled effectively. Further research on learning curves and the financial implications of increased operating time can allow training programs to be developed to help doctors familiarise themselves with robotic platforms and improve surgical workflows.

In addition, future studies should evaluate the clinical significance of improved mechanical alignment, particularly in relation to functional outcomes, implant survival and revision rates. To achieve this, standardized definitions and clear timepoints are necessary. Furthermore, reporting the absolute deviation of HKA is a more intuitive metric that can address the issues surrounding the directionality of postoperative HKA angle.

## Conclusions

In conclusion, RATKA has been shown to be better at improving postoperative mechanical alignment but is associated with a significantly longer operating time. It has demonstrated a good safety profile, alongside having comparable intraoperative blood loss to conventional techniques. In addition, it neither prolongs nor reduces hospital stay. However, it should be considered that improved radiographic alignment does not necessarily translate into superior long-term outcomes such as implant survival or reduced revision rates, as the included studies in this review were not designed to evaluate these outcomes. 

Further studies with standardized outcomes entailing longer follow-up periods are required to evaluate the factors affecting operating time and the clinical significance of improved mechanical alignment. This can help address the gap in duration of surgery by improving surgeon training programs and reducing the steep learning curve. Furthermore, future studies analyzing the cost-effectiveness of RATKA can help guide future technological innovation.
